# Transcriptional factor OmpR positively regulates prodigiosin biosynthesis in *Serratia marcescens* FZSF02 by binding with the promoter of the prodigiosin cluster

**DOI:** 10.3389/fmicb.2022.1041146

**Published:** 2022-11-17

**Authors:** Xianbo Jia, Ke Zhao, Fangchen Liu, Junjie Lin, Chenqiang Lin, Jichen Chen

**Affiliations:** ^1^Institute of Soil and Fertilizer, Fujian Academy of Agricultural Sciences, Fujian Key Laboratory of Plant Nutrition and Fertilizer, Fuzhou, China; ^2^College of Resources and Environment, Fujian Agriculture and Forestry University, Fuzhou, China; ^3^College of Life Sciences, Fujian Agriculture and Forestry University, Fuzhou, China

**Keywords:** *Serratia marcescens*, prodigiosin, OmpR, two-component system, regulatory mechanism

## Abstract

Prodigiosin is a promising secondary metabolite mainly produced by *Serratia marcescens*. The production of prodigiosin by *S. marcescens* is regulated by different kinds of regulatory systems, including the EnvZ/OmpR system. In this study, we demonstrated that the regulatory factor OmpR positively regulated prodigiosin production in *S. marcescens* FZSF02 by directly binding to the promoter region of the prodigiosin biosynthesis cluster with a *lacZ* reporter assay and electrophoretic mobility shift assay (EMSA). The binding sequence with the *pig* promoter was identified by a DNase I footprinting assay. We further demonstrate that OmpR regulates its own expression by directly binding to the promoter region of *envZ*/*ompR*. For the first time, the regulatory mechanism of prodigiosin production by the transcriptional factor OmpR was revealed.

## Introduction

Prodigiosin, an important secondary metabolite produced by *S. marcescens* and some other bacteria, is of particular interest for its potential applications, including various pharmacological activities, food colorants, and potential sunscreens (Stankovic et al., 2014; Darshan and Manonmani, 2015). In the genus *Serratia*, biosynthesis of prodigiosin is regulated not only by external factors, including temperature, pH and medium composition (Paul et al., 2020) but also by various genes (Williamson et al., 2006). Nearly 30 genes have been reported to be involved in prodigiosin biosynthesis in *S. marcescens* (Pan et al., 2021), and more studies should be carried out to search for new regulatory genes and uncover the complex regulatory mechanisms of this secondary metabolite.

The two-component system is a family of signal transduction proteins reported to be present in all types of life (Stock et al., 1999; Scharf, 2010; Papon and Stock, 2019). In bacteria, the classical two-component system consists of a sensor protein and a regulatory protein (Yuchuan et al., 2019). Sensor proteins respond to chemical or physical signals by phosphorylating regulatory proteins, and phosphorylated regulatory proteins can regulate the expression levels of downstream genes by binding to certain DNA sequences (Groisman, 2016). Two-component systems regulate many processes of bacteria, such as adaptation to environmental changes: osmolarity (Boyce et al., 2016), temperature (Dhiman et al., 2015; Najnin et al., 2016), oxygen (Dixon, 1998; Wright et al., 2018), regulation of developmental pathways, and behaviors, such as sporulation (Sarwar and Garza, 2015), biofilm formation (Lai et al., 2005), quorum sensing (Kruppa et al., 2004), regulation of secondary metabolite biosynthesis (Sola-Landa et al., 2003), virulence (Beier and Gross, 2006), and antibiotic resistance (Bhagirath et al., 2019; Tierney and Rather, 2019). Biosynthesis of prodigiosin was also regulated by different types of two component systems in various *Serratia* strains, including PigQ/W and PhoB/PhoR in *Serratia* 39,006 (Fineran et al., 2005; Gristwood et al., 2009); EepR/EepS in *S. marcescens* CMS376, *S. marcescens* K904, and *S. marcescens* Nima (Stella et al., 2015); RssB/RssA in *S. marcescens* CH-1 (Horng et al., 2010); and CpxR/A in *S. marcescens* FS14 (Qiu et al., 2021).

The two component system EnvZ/OmpR is an important signal transduction system in bacteria responding to various environmental stress and growth conditions (Qin et al., 2001). We have previously demonstrated that when *envZ* or *ompR* was knocked out, *S. marcescens* FZSF02 lost its prodigiosin biosynthesis ability (Jia et al., 2021), and OmpR was also recently found to control prodigiosin biosynthesis in *S. marcescens* JNB5-1 (Pan et al., 2022). However, the regulatory mechanism of the two-component EnvZ/OmpR system on prodigiosin production is still unknown.

In this study, with *LacZ*-reporter studies and an electrophoretic mobility shift assay (EMSA), we demonstrated that OmpR positively regulated prodigiosin biosynthesis by directly binding to the promoter region of the prodigiosin biosynthesis gene cluster. We also found that OmpR can regulate its own expression level by binding the promoter of the EnvZ/OmpR genes.

## Materials and methods

### Bacterial strains, plasmids, and culture conditions

The bacterial strains and plasmids used in this study are listed in Supplementary Tables S1, S2. *Serratia marcescens* FZSF02 and its related mutants were incubated in lysogeny broth (LB) at 28°C and 180 rpm. *E. coli* DH5a and *E. coli* Rosetta (DE3) were cultured in LB medium at 37°C and 220 rpm. The final concentrations of antibiotics used in this study were as follows: 100 mg/L ampicillin, 100 mg/L kanamycin, and 50 mg/L chloramphenicol.

### Construction of the in-frame deletion mutant and complementary strains

FZSF02∆*ompR* and FZSF02∆*envZ* were constructed in our previous study (Jia et al., 2021). FZSF02∆*envZ*∆*ompR* was also constructed with the homologous recombination method (Jia et al., 2021). Complementary strains were constructed with the plasmid pRK415 as we have reported in our previous study (Jia et al., 2021). pRK415-*ompR*, pRK415-*envZ* and pRK415-∆*envZ*∆*ompR* were transformed into FZSF02∆*ompR*, FZSF02∆*envZ*, and FZSF02∆*envZ*∆*ompR,* respectively, to construct the complementary strains. The primers used in these experiments are listed in Supplementary Table S2.

### *lacZ* reporter assays

The *pig* promoter and *ompR* promoter were inserted upstream of *lacZ* in the plasmid pTOPO-*lacZ*-Cmr to construct the plasmids pTOPO-*Pig*pro-*lacZ*-Cmr and pTOPO-OmpRpro-*lacZ*-Cmr. The primers used in this experiment are listed in Supplementary Table S2. The constructed plasmids with the *Pig* promoter and *ompR* promoter were transformed into the wild-type strain *S. marcescens* FZSF02 and the *ompR*-knockout strain FZSF02∆ompR, respectively. The plasmids pTOPO-*Pig*pro-Cmr and pTOPO-*ompR*pro-Cmr were also transformed into *S. marcescens* FZSF02 and FZSF02∆ompR as controls. For liquid β-galactosidase assays, constructed strains were cultured at 28°C and 180 rpm for 16 h, and enzyme activities were measured in sonicated extracts according to the method described by Pardee et al. (1959).

### Electrophoretic mobility shift assay

The coding sequence of *ompR* was cloned into pEASY®-Blunt E2 (TransGen Biotech, Beijing, China). The plasmid pEASY®- Blunt E2-*ompR* was transformed into *E. coli* Rosetta (DE3). The DNA fragments containing the *pig* promoter (406 bp) and *ompR* promoter (257 bp) were cloned into the pTOPO-Blunt simple vector (Aidlab, China), respectively, and then promoter probes were obtained through polymerase chain reaction (PCR) with primers M13F and M13R labeled with Cy5.5 at the 5′ end. The *Pig*pro probe and *ompR*pro probe were 556 and 407 bp, respectively. Electrophoretic mobility shift assay (EMSA) was carried out with an EMSA/Gel-Shift kit (Beyotime, Shanghai, China). The purified probe and protein were mixed with EMSA/Gel-Shift binding buffer (5×), and a total of 10 μl of the reaction system was supplied with distilled water and incubated for 30 min at 28°C. 6% native PAGE was prepared as the kit protocol described, and the reaction mixture was loaded onto the PAGE. Electrophoresis was performed at 60 V for 3 h in 0.5× TBE buffer, and the gels were exposed to an Odyssey CLx (LI-COR® Biosciences).

### DNase I footprinting assay

The *pig* promoter probe was prepared by polymerase chain reaction (PCR) with primers M13F and M13R (labeled with Hex). The DNase I foot-printing assay was carried out as described by Shi et al. (2017).

## Results

### OmpR activates the transcription level of the *pig* gene cluster

**Figure 1:**
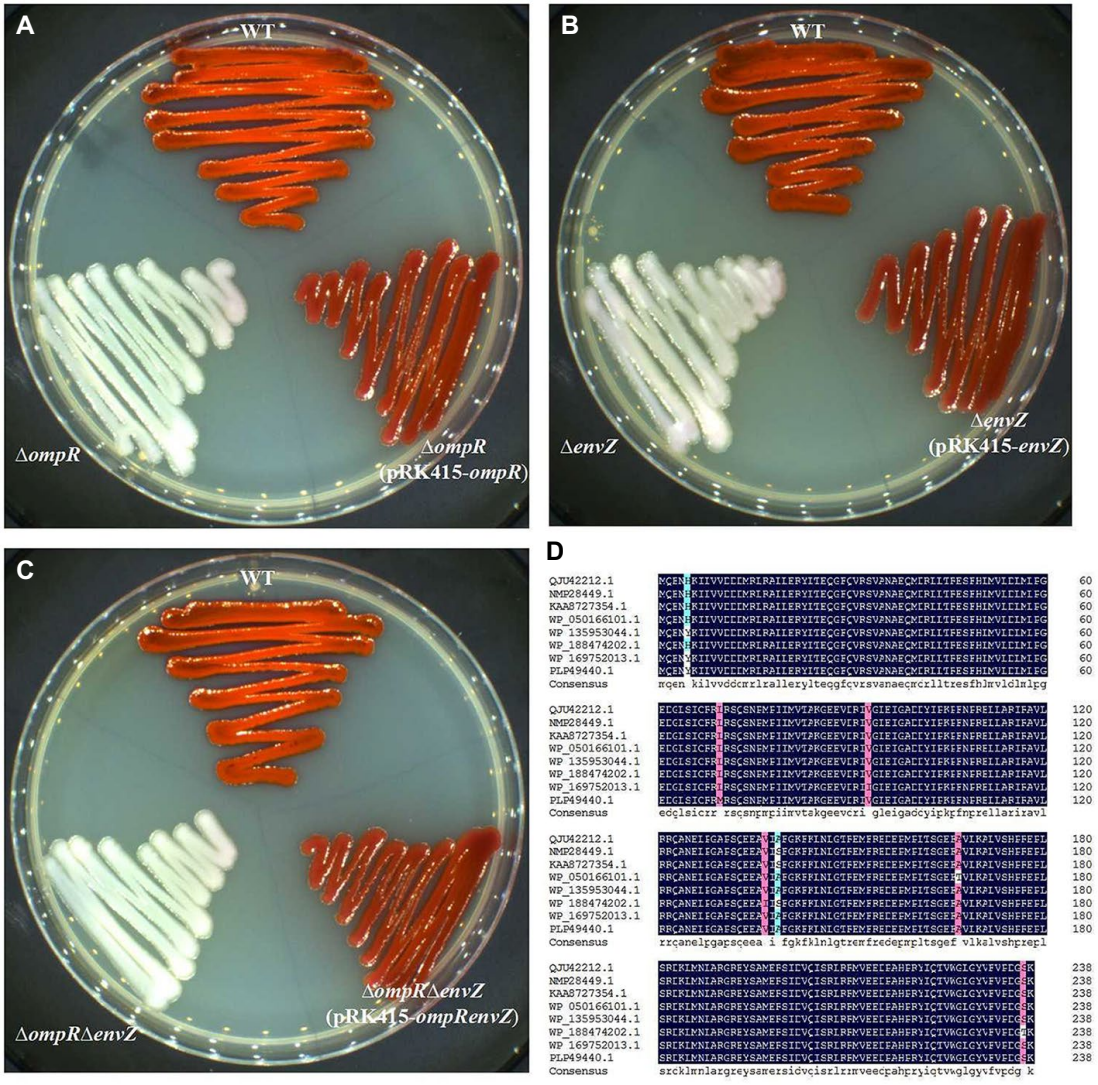
EnvZ/OmpR two-component system and its effect on the prodigiosin-producing ability of *S. marcescens* FZSF02. **(A)** Prodigiosin-producing ability of strain WT (wild-type strain of *S. marcescens* FZSF02), and strain ∆*ompR* (in frame deletion of *ompR* of FZSF02), complementary strain ∆*ompR* (pRK415-*ompR*). **(B)** Prodigiosin-producing ability of strain WT (wild-type strain of *S. marcescens* FZSF02), ∆*envZ* (in frame deletion of *envZ* of FZSF02) and complementary strain ∆*envZ* (pRK415-*envZ*). **(C)** Prodigiosin-producing ability of strain WT (wild-type strain of *S. marcescens* FZSF02), strain ∆*ompR*∆*envZ* (in frame deletion of *envZ* and *ompR* of FZSF02) and complementary strain ∆*ompR*∆*envZ* (pRK415-*ompRenvZ*). All strains were incubated on LB agar plates at 27°C for 48 h. **(D)** Multiple sequence alignment of OmpR homologies to analyze its high conservation. Sequences chosen for this analysis were from *S. marcescens* FZSF02 (QJU42212.1), *R. aceris* (NMP28449.1), *E. americana* (KAA8727354.1), *Y. enterocolitica* (WP_050166101.1), *C. youngae* (WP_135953044.1), *H. psychrotolerans* (WP_188474202.1), *E. coli* (WP_169752013.1), and *K. pneumoniae* (PLP49440.1).

We have previously demonstrated that gene deletion of *ompR* (GenBank: QJU42212.1) or *envZ* (GenBank: QJU42211.1) would result in loss of prodigiosin producing ability in *S. marcescens* FZSF02; transcription levels of *pigA* also down-regulated significantly when *ompR* or *envZ* was knocked out assayed by qPCR (Jia et al., 2021). The effect of OmpR and EnvZ on prodigiosin-producing ability was further demonstrated by knockout *ompR* and *envZ*, double knockout of *envZ* and *ompR* in this study; gene deletion strains all lost prodigiosin-producing ability, prodigiosin-producing ability restored in complementary strain (Figures 1A–C). OmpR was very conserved in amino acid sequences among many Gram-negative bacteria, such as *Rouxiella aceris, Ewingella americana, Yersinia enterocolitica, Citrobacter youngae, Hafnia psychrotolerans, Escherichia coli* and *Klebsiella pneumoniae* (Figure 1D). Many studies have demonstrated that OmpR influences a wide variety of cellular processes in *E. coli*, *Salmonella* sp. and *Shigella* sp., as well as pathogenic species of *Yersinia* sp. (Jaworska et al., 2021), but the influence of secondary metabolite biosynthesis by OmpR has rarely been reported. We have previously demonstrated that OmpR can positively regulate the production of the secondary metabolite prodigiosin in *S. marcescens* FZSF02, but the regulatory mechanism was unknown.

**Figure 2:**
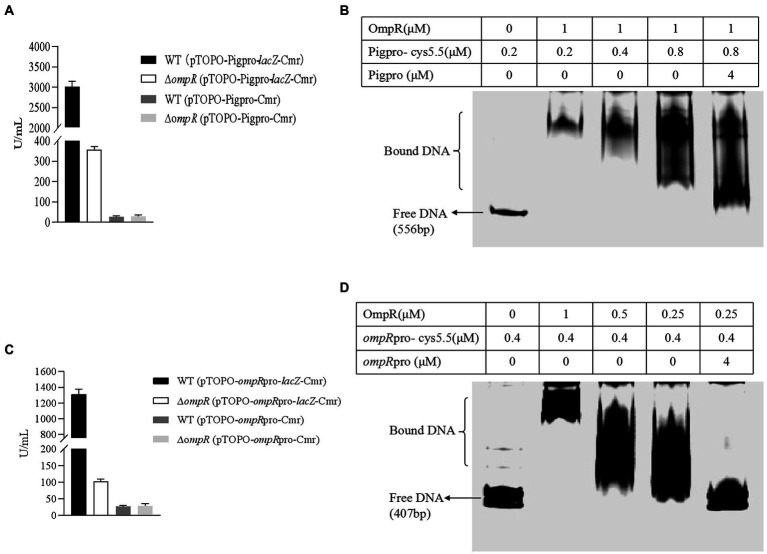
OmpR regulates the prodigiosin-producing ability of FZSF02 directly. **(A)** Analysis of β-galactosidase activity of ∆ompR (pTOPO-*Pig*pro-*lacZ*-Cmr) and WT (pTOPO-*Pig*pro-*lacZ*-Cmr) harboring the *Pig*pro-*lacZ* reporter fusion. WT (pTOPO-*Pig*pro-Cmr) and ∆ompR (pTOPO-*Pig*pro-Cmr) were constructed as controls. **(B)** EMSA for OmpR protein binding to the promoter of the *pig* cluster. **(C)** Analysis of β-galactosidase activity of ∆ompR (pTOPO-*ompR*pro-*lacZ*-Cmr) and WT (pTOPO-*ompR*pro-*lacZ*-Cmr) harboring the *ompR*pro-*lacZ* reporter fusion. WT (pTOPO-*ompR*pro-Cmr) and ∆ompR (pTOPO-*ompR*pro-Cmr) were constructed as controls. **(D)** EMSA for assay OmpR protein binding ability to the promoter of *envZ*/*ompR*.

To further test whether the regulation of prodigiosin biosynthesis by OmpR is at the transcriptional level, the β-galactosidase activity of FZSF02 and FZSF02∆*ompR* was assayed when the *lacZ* gene was under control by the *pig* cluster promoter. The results showed that the β-galactosidase activity of FZSF02∆*ompR* (pTOPO-*Pig*pro-*lacZ*-Cmr) decreased by 88.5% compared with that of the wild-type strain FZSF02 WT (pTOPO-*Pig*pro-*lacZ*-Cmr; Figure 2A), and almost no β-galactosidase activity was tested in the control group of WT (pTOPO-*Pig*pro-Cmr) and ∆*ompR* (pTOPO-*Pig*pro-Cmr; Figure 2A). This result indicates that OmpR directly or indirectly activates the transcription level of the *pig* gene cluster, and influences prodigiosin synthesis in strain FZSF02.

### OmpR activates the transcription level of the *pig* gene cluster by directly binding to its promoter sequence

**Figure 3:**
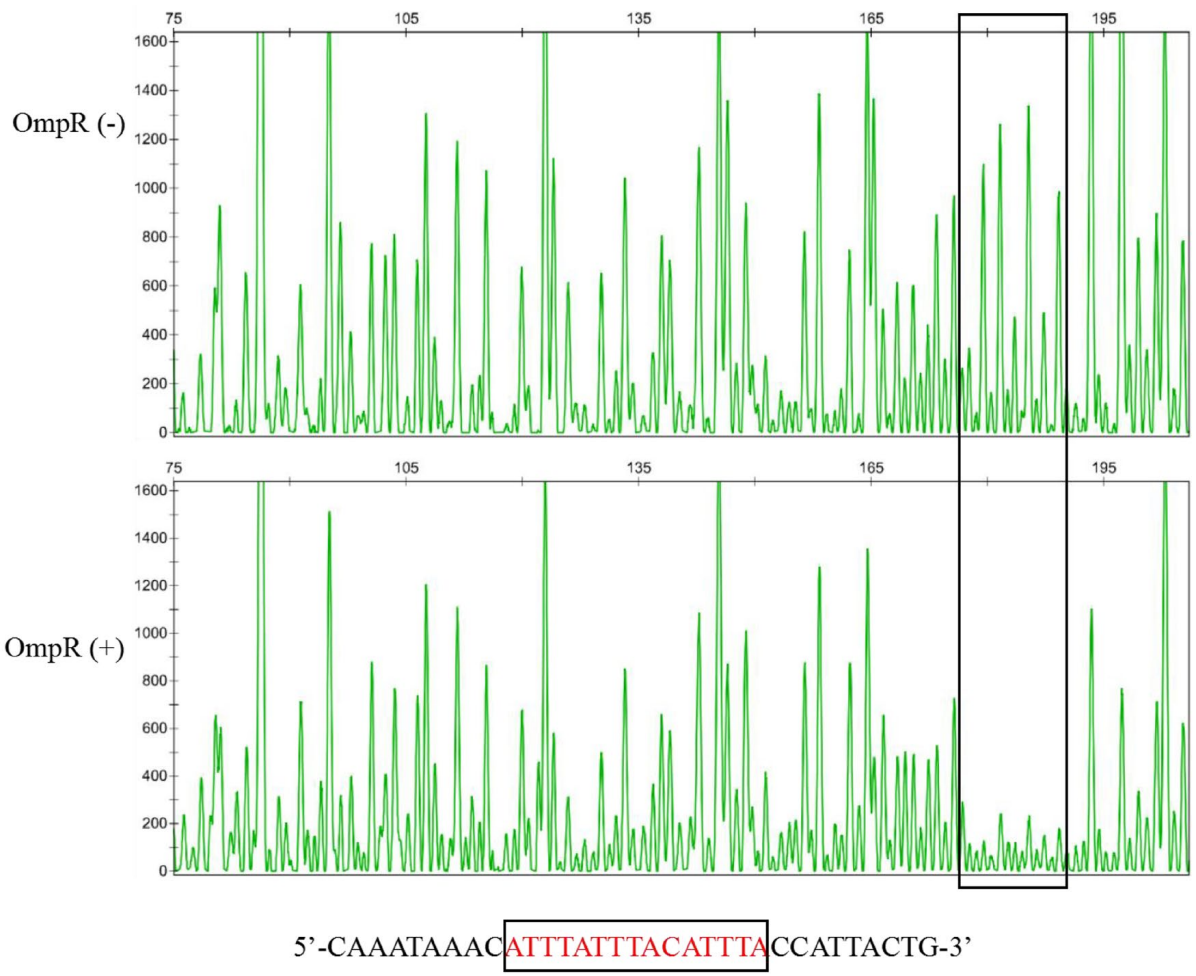
Confirmation of the proposed binding sequence of OmpR with the *pig* cluster promoter by DNaseI foot-printing assay. The OmpR (−) group indicates that the promoter probe was not incubated with OmpR protein before treatment with DNase I, and the OmpR (+) group indicates that the promoter probe was incubated with OmpR protein before treatment with DNase I.

To study whether OmpR regulates the expression of the *pig* gene cluster by binding with its promoter directly, EMSA was used to detect the binding ability between OmpR and the *pig* gene cluster promoter sequence. The results showed that OmpR can bind with the probe prepared with the prodigiosin cluster promoter sequence (Figure 2B). A DNase I footprinting assay showed that the proposed binding sequence of OmpR on the *pig* promoter was 5′CATTTATTTACATTTAC3′ (Figure 3), which located on −103 bp to −86 bp relative to the A of the ATG start codon of *pigA. Pig* promoter sequence is between *pigA* (QJU38817.1) and *cueR* (QJU38818.1) on the genome of FZSF02.

### OMPR activates its own expression level

To test whether the autoregulation of OmpR exists in *S. marcescens* FZSF02, the β-galactosidase activity of FZSF02 and FZSF02∆*ompR* was assayed when the *lacZ* gene was first controlled by the *ompR* promoter. The results showed that the β-galactosidase activity of FZSF02∆*ompR* (100 U/ml) decreased by 92.3% compared with that of the wild-type strain FZSF02 (1,300 U/ml; Figure 2C). This finding demonstrates that OmpR can activate its own expression level.

### OmpR can directly bind to the *envZ*/*ompR* promoter

To test whether the activation of OmpR on its own expression is performed by binding with the *envZ*/*ompR* promoter, EMAS was used to examine the binding ability between OmpR and the *envZ*/*ompR* promoter sequence. The results showed that when the *envZ*/*ompR* promoter sequence was used as a probe, OmpR could bind with the labeled probe (Figure 2D). The addition of unlabeled probe can compete with the labeled probe, which further demonstrates the binding ability.

## Discussion

Prodigiosin was a kind of bacterial secondary metabolites produced mainly by many *S. marcescens* strains. Various regulating genes involved in prodigiosin biosynthesis have been found in the past two decades, but new regulators, such as RcsB (Pan et al., 2021), CpxA/R (Qiu et al., 2021) and Fnr (Sun et al., 2021), have still been reported continuously. Research of the these genes may help to uncover the regulatory mechanism behind prodigiosin biosynthesis in *S. marcescens*.

EnvZ/OmpR is known to control motility (Prüß, 2017), intracellular survival (Du et al., 2022), antibiotic resistance (Ko and Choi, 2022), virulence (Tipton and Rather, 2017), and other characteristics of different bacterial strains, but few studies have reported the role of EnvZ/OmpR in *S. marcescens.* We have demonstrated previously that mutation of *ompR* or *envZ* would cause the loss of prodigiosin producing ability in *S. marcescens* FZSF02 and confirmed that the two-component system EnvZ/OmpR was a newly found system that can regulate prodigiosin biosynthesis (Jia et al., 2021). In this study, the regulatory function of EnvZ/OmpR on prodigiosin biosynthesis was further confirmed by gene deletion and complementation (Figure 1C). For the EnvZ/OmpR system, OmpR was reported to play the role by binding to the gene promoters and regulating the expression of other genes (Wang et al., 2021). *LacZ* reporter assays and EMSA assay in this study also showed OmpR regulate prodigiosin biosynthesis by directly binding to the promoter region of *pig* cluster (Figures 2A,B). The binding region of the OmpR was identified as 5′CATTTATTTACATTTAC3′ (Figure 3) by a DNase I footprinting assay. The binding sequence showed 45% identity to the *E. coli* consensus sequence (5′TTTTACTTTTGTAACATAT3′; Maeda et al., 1991) and 55% identity to that of *Y. enterocolitica* (5′ATTTATTGATGGTAACAATT3′; Nieckarz et al., 2020).

Many two-component systems regulating proteins can autoregulate their own expression by binding to their promoters, and this kind of feedback allows the regulatory functions of the system to be more flexible (Groisman, 2016). For the EnvZ/OmpR two-component system, autoregulation differs among different strains; it exists in *Salmonella enterica* (Bang et al., 2002; Cameron and Dorman, 2012) but not in *E. coli* (Ochman and Wilson, 1987; Doolittle et al., 1996) and *Acinetobacter baumannii* (Tipton and Rather, 2017). The results of the *LacZ* reporter assay (Figure 2C) and EMSA assay (Figure 2D) indicated that OmpR can bind to the promoter region of *envZ*/*ompR* and promote the expression of OmpR and EnvZ.

**Figure 4:**
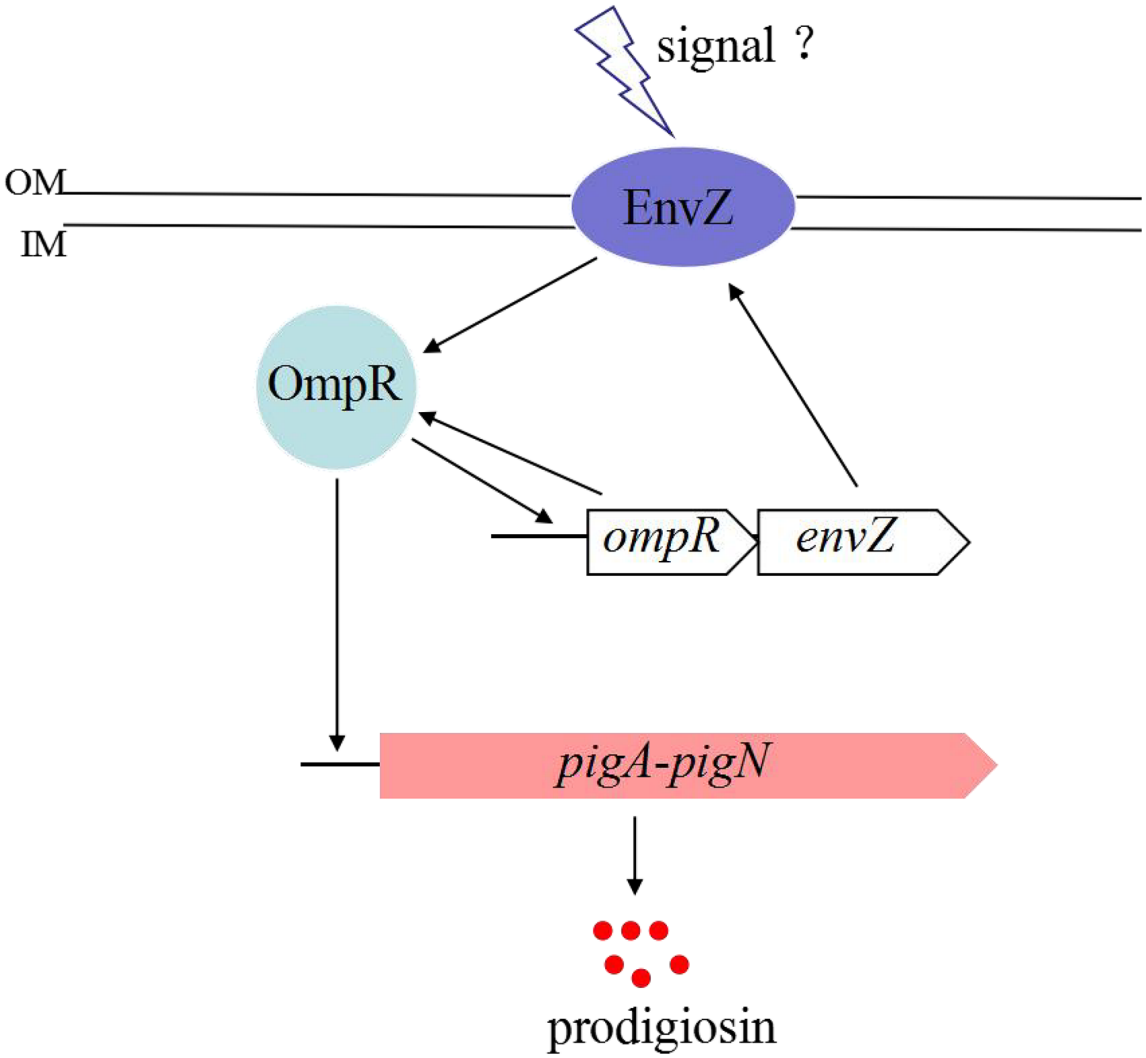
Proposed model of OmpR involvement in the regulatory mechanism of prodigiosin biosynthesis in *S. marcescens* FZSF02.

Based on the above results, we proposed that the regulatory mechanism of the two-component EnvZ/OmpR system on prodigisin biosynthesis was probably as follows (Figure 4): Some unknown factors induced the expression of EnvZ and OmpR, and OmpR was then phosphorylated by EnvZ (Cai and Inouye, 2002). Phosphorylated OmpR activated the expression of more EnvZ and OmpR by binding with the *envZ*/*ompR* promoter. When the concentration of OmpR reached a certain level, the *pig* gene cluster promoter persistently bound with OmpR, and genes involved in prodigiosin biosynthesis were highly expressed at the transcriptional level.

Although we have previously found that when ompR was knocked out, *S. marcescens* FZSF02 lost the prodigiosin biosynthesis ability (Jia et al., 2021), in this study, the proposed regulatory mechanism of OmpR on prodigiosin biosynthesis was demonstrated. EnvZ/OmpR was demonstrated to be a new two-compound system that can directly positively regulate prodigiosin production in *S. marcescens* FZSF02.

## Data availability statement

The original contributions presented in the study are included in the article/Supplementary material, further inquiries can be directed to the corresponding author.

## Author contributions

XJ and JC designed the study, wrote the manuscript, and analyzed the results. XJ, FL, JL, CL, and KZ performed the experiments. All authors contributed to the article and approved the submitted version.

## Funding

This study was supported by the Exploration Program of Fujian Academy of Agricultural Sciences (ZYTS202217), Scientific Research in the Public Interest of Fujian Province (2020R1025003 and 2021R1025002), Natural Science Foundation of Fujian Province of China (2021J01480), Chinese National Natural Science Foundation (31800068), the Special Program for Extension Research of National Natural Science Foundation of Fujian Academy of Agricultural Sciences (AGY2018-1), and Science and Technology Innovation Team Program of Fujian Academy of Agricultural Sciences (CXTD2021002-3).

## Conflict of interest

The authors declare that the research was conducted in the absence of any commercial or financial relationships that could be construed as a potential conflict of interest.

## Publisher’s note

All claims expressed in this article are solely those of the authors and do not necessarily represent those of their affiliated organizations, or those of the publisher, the editors and the reviewers. Any product that may be evaluated in this article, or claim that may be made by its manufacturer, is not guaranteed or endorsed by the publisher.
