# Supplementary material for: Transcriptional factor OmpR positively regulates prodigiosin biosynthesis in Serratia marcescens FZSF02 by binding with the promoter of the prodigiosin cluster
Source: Front Microbiol. 2022 Nov 17;13:1041146. doi: 10.3389/fmicb.2022.1041146 (PMC9712742; doi:10.3389/fmicb.2022.1041146)
Supplement: Supplementary file 1 [file Data_Sheet_1.docx]

| Strains | Description | Source |
| --- | --- | --- |
| ***S.marcescens* FZSF02** |  |  |
| WT | Wild type strain of FZSF02 | This study |
| ∆*ompR* | *ompR* gene knockout | This study |
| ∆*envZ* | *envZ* gene knockout | This study |
| ∆*ompR*∆*envZ* | *Double knocked out of ompR* and *envZ* | This study |
| WT (pTOPO-Pigpro-Cmr) | WT carries pTOPO-Pigpro-Cmr | This study |
| ∆*ompR* (pTOPO-Pigpro-Cmr) | ∆*ompR* carries pTOPO-Pigpro-Cmr | This study |
| WT (pTOPO-Pigpro *-lacZ-*Cmr) | WT carries pTOPO-Pigpro-*lacZ*-Cmr | This study |
| ∆*ompR* (pTOPO-Pigpro *-lacZ-*Cmr) | ∆*ompR* carries pTOPO-Pigpro-*lacZ*-Cmr | This study |
| WT (pTOPO-OmpRpro *-lacZ-*Cmr) | WT carries pTOPO-OmpRpro-*lacZ*-Cmr | This study |
| ∆*ompR* (pTOPO-OmpRpro*-lacZ-*Cmr) | ∆*ompR* carries pTOPO-OmpRpro-*lacZ*-Cmr | This study |
| WT (pTOPO-*ompR*pro-Cmr) | WT carries pTOPO-*ompR*pro-Cmr | This study |
| ∆*ompR* (pTOPO-*ompR*pro-Cmr) | ∆*ompR* carries pTOPO-*ompR*pro-Cmr | This study |
| ***E.coli DH5α*** |  |  |
| DH5*α* (pEASY-*ompR*） | DH5*α* carries pEASY-*ompR* | This study |
| DH5*α (*pTOPO-Pigpro-Cmr) | DH5*α* carries pTOPO-Pigpro-Cmr | This study |
| DH5*α* (pTOPO-Pigpro*-lacZ-*Cmr) | DH5*α* carries pTOPO-Pigpro*-lacZ-*Cmr | This study |
| DH5*α* (pTOPO-OmpRpro*-lacZ-*Cmr) | DH5*α* carries pTOPO-OmpRpro-*lacZ*-Cmr | This study |
| DH5*α* (pTOPO-*ompR*pro*-*Cmr) | DH5*α* carries pTOPO-ompRpro-Cmr | This study |
| ***E.coli BL21*** |  |  |
| BL21（pEASY-*ompR*） | BL21 carries pEASY-*ompR* | This study |

**Table S1 Strains used in this study**

**Table S2 Plasmids used in this study**

| **Plasmid** | **Description** | **Soure** |
| --- | --- | --- |
| pTOPO-Blunt simple vector | CV22-Zero Background pTOPO-Blunt Simple Cloning Kit | Aidlab |
| pTOPO-Cmr | Cmr resistance gene was added to pTOPO vector | This study |
| pTOPO-Pigpro-*lacZ*-Cmr | Pigpro-*lacZ* fragment is connected to pTOPO-Cmr linearization vector | This study |
| pTOPO-*lacZ*-Cmr | *lacZ* fragment is connected to pTOPO-Cmr linearization vector | This study |
| pTOPO-Pigpro-Cmr | The promoter of Pig gene cluster (Pigpro) was linked by pTOPO-Cmr | This study |
| pTOPO-OmpRpro-*lacZ-*Cmr | OmpRpro fragment was ligated on the basis of pTOPO-*lacZ*-Cmr recombinant plasmid | This study |
| pTOPO-*ompR*pro*-*Cmr | The promoter of *ompR* (*ompR*pro) was linked by pTOPO-Cmr | This study |
| pEASY®- Blunt E2 | pEASY®- Blunt E2 Expression Kit | TransGen Biotech, Beijing, China |
| pEASY-E2-*ompR* | The *ompR* gene fragment was linked to the pEASY-Blunt E2 plasmid | This study |
| pRK415-*envZ* | *envZ* gene was linked to the pRK415 plasmid | (Jia et al. 2021) |
| pRK415-*ompR* | *ompR* gene was linked to the pRK415 plasmid | (Jia et al. 2021) |
| pRK415 | Expression vector | Laboratory collection |

**Table S3 Primers used in this experiment**

| Primers | Sequences ( 5'→3') |  |
| --- | --- | --- |
| OmpRproF | TGTAAAACGACGGCCAGTCCCGACAGCTGAACGCCACCGGGC | For OmpR promoter amplification |
| OmpRproR | AGCTCCGGATCCGGTCATGGTATTACTCCCAAAGGCTTTATT | For OmpR promoter amplification |
| PigproF | TGTAAAACGACGGCCAGTTTTTTCCTCCGGAATGCTCCTGC | For pig cluster promoter amplification |
| PigproR | AGCTCCGGATCCGGTCATGACGAACTCCGCCATCGGGTTGA | For pig cluster promoter amplification |
| LacZF | ATGACCGGATCCGGAGCTTGGCT | For *lacZ* amplification |
| LacZR | TTATTTTTGACACCAGACCAACT | For *lacZ* amplification |
| M13F | TGTAAAACGACGGCCAGT | For pigpro and ompRpro probe [amplification](javascript:void(0);) |
| M13R | CAGGAAACAGCTATGACC | For pigpro and ompRpro probe [amplification](javascript:void(0);) |
| ExompRF | ATGCAAGAGAATCATAAGAT | For recombinant expressing OmpR in *E coli* |
| ExompRR | TGCCTTACTGCCGTCCGGGA | For recombinant expressing OmpR in *E coli* |
| KF1 | ATGCAAGAGAATCATAAGATCCTG | For in frame deletion of *envZ* and *ompR* |
| KR1 | CATCGATGATGGTTGAGAGTCGGCGCCGATTTCCAGCCCCA | For in frame deletion of *envZ* and *ompR* |
| KF2 | CTCGATGAGTTTTTCTAAAGATGGCGTCGGGCGTCAAGCAGC | For in frame deletion of *envZ* and *ompR* |
| KR2 | TCTCCATCGGCAACGGAATATACG | For in frame deletion of *envZ* and *ompR* |
| KanF | TCTCAACCATCATCGATGAATTGT | For in frame deletion of *envZ* and *ompR* |
| KanR | TTAGAAAAACTCATCGAGCATCAA | For in frame deletion of *envZ* and *ompR* |
| EOF | ACAGTAATACAAGGGGTGTTATGCAAGAGAATCATAAGATC | For complementary expression of *envZompR* |
| EOR | TCAGGCGTTTTCCCTGGCCGTT | For complementary expression of *envZompR* |
